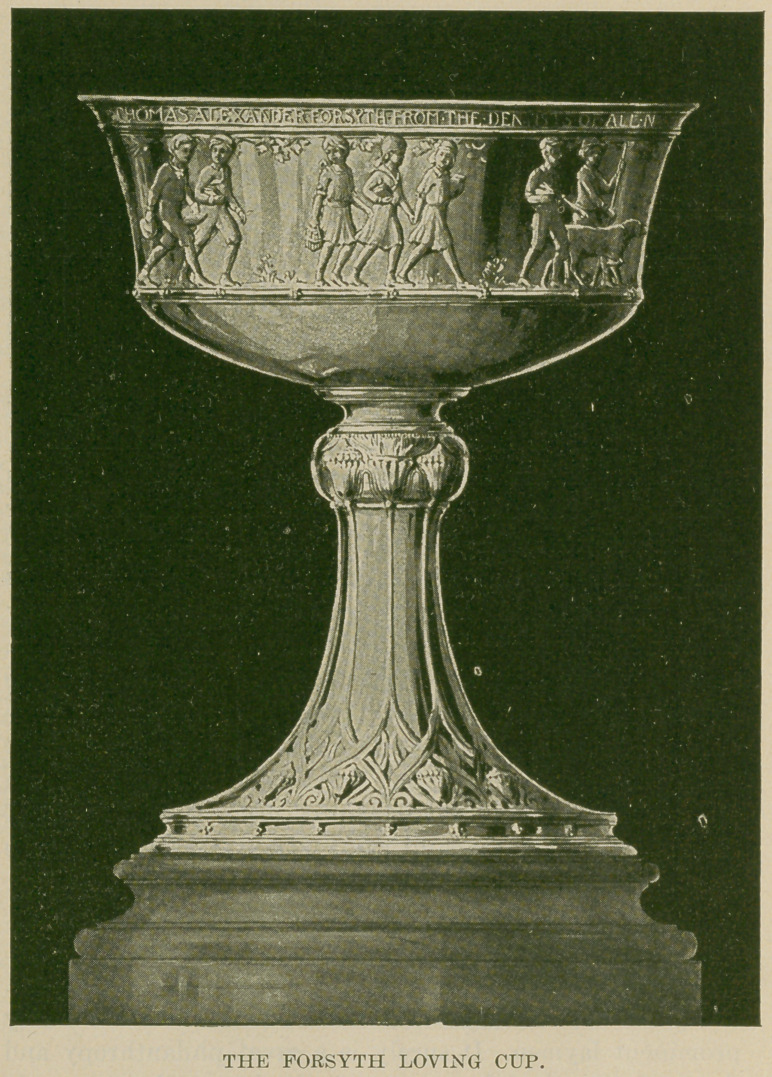# Event and Comment

**Published:** 1917-01

**Authors:** 


### DENTAL REGISTER

Vol. LXXI JANUARY, 1917 No. 1

### EVENT AND COMMENT

THE FORSYTH LOVING CUP. We are printing in this issue a picture of the cup presented to Thomas Alexander Forsyth by the members of the profession in this country and Canada. It will be remembered that the editor of *Oral Hygiene,* Dr. Belcher, opened a popular subscription for this purpose, and more than one thousand dollars were collected and the cup was designed and made by Tiffany & Co. of New York. It is a beautiful piece of work. On the upper portion of the bowl is an inscription to Mr. Forsyth from dentists all over the world in appreciation of the gift of this great institution by the Forsyth family for the amelioration of children suffering from disease of the teeth. Many figures ornament the sides of the bowl, symbolizing child life. The presentation is made a formal occasion with a banquet, with Dr. L. L. Barber, president of the National Dental Association presiding; Dr. W. H. G. Logan, of Chicago, as toast master, and Dr. H. E. Friesell, of Pittsburg, making the presentation speech. Delegates from every state were invited, besides many prominent laymen. It surely is a great philanthropy and the profession is doing a most creditable act in thus publicly recognizing it, and expressing its appreciation of the magnificent manner in which the Forsyths have carried out this scheme of benevolence. We confidently believe this will prove to be one of the greatest factors in pre-

THE FORSYTH LOVING CUP.

ventive medicine that has ever been undertaken. That the benefactors and those having charge of its conduct may have wisdom sufficient for its conduct, is the prayer of humanity.

OFFICERS OF OHIO STATE DENTAL SOCIETY FOR 1917. The following officers were elected at the last meeting in December: President, Dr. F. M. Casto, of Cleveland; First Vice President, Dr. Z. N. Wright, of Dayton; Second Vice President, Dr. W. H. Hayden, of Youngstown; Drs. L. L. Barber, H. C. Brown, J. R. Callahan, and L. E. Custer, were elected as delegates to the National Dental Society. The next meeting will be held in Cleveland.

MEETING OF THE OHIO STATE DENTAL SOCIETY. The fifty-first annual meeting was held this year in Dayton, and was very well attended, and all its sessions were most interesting. President Way presided with dignity and kept the order of the program consistently. The president's address was a strong plea for an oral hygiene propaganda in the state, as the most hopeful method of controlling the progress of dental caries. He also strongly endorsed the research work of the national society and urged its continued and increased support by the profession.

Dr. W. E. Cummer, of Toronto, Canada, gave a lecture on the opportunity and necessity of partial dentures as a logical substitute for bridge work. He presented his ideas of retention which are unique as applied to partial denture, especially metal dentures. He subsequently showed by a clinic his technical methods of construction, which have been published recently in several of the dental journals.

Dr. Howard R. Raper, of Indianapolis, gave a very entertaining lecture on radiography. He took the position that there was formally some excuse for bad root canal treatment, but that the dentist of today who did not know whether he was doing this work adequately or not, was not meeting his obligations to his patents. He incidentally contended that too many teeth were crowned that might better have been filled, because of the fact that the average dentist makes better fillings than he does crowns, and that root canal complications are not so liable to bring secondary complications.

Dr. Martin Fischer, Professor of Physiology in the University of Cincinnati, gave an interesting address, on oral sepsis as related to systemic diseases. He gave a very lucid explanation of systemic infection from local leasions, and arraigned the dental profession for adhering to certain forms of dental treatments that had been shown to be most hazardous to general health. He thought it better to lose a tooth than die of appendicitis or be a life cripple from rheumatism. He urged a closer study of local and general pathologic conditions by both the dental and medical professions.

Dr. M. J. Rosenau, Professor of Preventive Medicine in Harvard University, gave an interesting and instructive lecture on the principles of Preventive Medicine as related to dentistry. He said that the larger percentage of infectious diseases were either located in the mouth or had their origin there. There are so many ways in which diseases of the mouth, nose and throat can be transmitted that it would seem almost impossible to prevent the spread of disease so long as our social relations are so intimate. Spitting sneezing, coughing, hand shaking, kissing, and even breathing are the common carriers of infection. It is practically impossible to counteract such influences unless the organs of the mouth, nose, throat, lungs, etc., are kept healthy, and people are made intelligent as to the dangers attending contact under unhealthy conditions. The dentist as well as the physician has a most important function in bringing about preventive medicine.

Dr. Harold D. W. Cross of the Forsythe Dental Infirmary, gave a very interesting lecture on the work of this great institution in carrying out its work for oral hygiene. With the facilities afforded by this great institution, it is possible to carry on work of this kind more advantageously than the practitioners, even in cities where there are similar facilities can hope to do. It will be interesting to keep tab on the statistics accumulated in this work as they will be invaluable as a basis for future efforts along this line.

Dr. Thadius Hyatt, of New York, sent his address, and it contains much that will be of value to any one who is devising plans for doing work in dental hygiene. Lectures, charts, etc., should be made up by experts and published so as to be available for workers in this field.

Dr. J. R. Callahan, of Cincinnati, gave a good practical address on disinfection and sterilization of dental instruments. He gave some striking illustrations of the inadequate sterilization as ordinarily practiced by dentists. He advocated heat sterilizers for instruments, and cotton used in canal work. His methods and ideals were undoubtedly correct scientifically, but we fear few dentists will be able to realize them in practice.

ORAL HYGIENE CONFERENCE. It seemed as though the whole meeting was pointed toward oral hygiene, and the climax was reached in a dinner at the hotel attended by about 400 dentists and physicians. This meeting was arranged by Dr. Sidney Rauh, of Cincinnati, and it took the form of short addresses by workers in this subject, particularly along the line of work in the schools, institutions and public gatherings.

The addresses were full of enthusiasm and helpful suggestions. It is hoped that the work of the State Oral Hygiene Committee will be greatly benefited by this meeting. In this connection, Dr. Rauh and his assistants have prepared an excellent museum illustrating dental diseases and methods of oral hygiene, which it is intended shall be at the disposal of the local and district societies throughout the state. It is put up in portable form and should be presented in every community in the state. It is planned to have this done if the funds can be provided for this purpose.

DR. WESTON A. PRICE, gave one of his characteristic addresses on the biological factors of disease, and made a statement of the progress of the work being done at the research laboratories. His address was illustrated with lantern slides and moving pictures, and was most intertaining and instructive. At the close of his address by a unanimous vote of the Society it was decided to give one dollar per member to the research work, and the annual dues were ordered increased to that extent.

CLINICS. There were not so many clinics as are often found on the programs of large conventions, but those given were on live subjects and by expert clinicians. Three clinics were given as demonstrated lectures, one on impression taking for dentures by Dr. R. E. Hall, one on orthodontia, by Dr. Victor H. Jackson, and one on conductive anesthesia by Dr. A. E. Smith, of Chicago. These clinics were all excellently presented and greatly appreciated. The other clinics were given by dividing the audience into equal sections and having the clinician move from one section to another, so that every one had a chance to see every clinic if he so desired. Six clinics were given in this manner, on these subjects, Acurately Fitted Gold Crowns, by Dr. L. Atkinson; Inlay Attachment for a Fixed Bridge, by Dr. A. J. Bush; Splint for Pyorrhetic Teeth, by Dr. G. S. Hershey; Partial Dentures, by Dr. W. E. Cummers; Radiography, by Dr. Wm. Doughty; Amalgam Restorations, by Dr. J. A. Dinwiddie.

Besides these, general clinics were given by Dr. M. Gruenbaum, on Removable Dentures; Dr. O. Miesse, on Construction of Richmond Bicuspid; Dr. P. A. Gould, on Gold Crown; Dr. H. V. Cattrell, on Prosthetics; Dr. L. T. Sauerbraun, on Special Trays for Impressions and Bites; Dr. H. C. Dean, on Hand Made Backing for Interchangeable Facings; Dr. C. C. Scott, Patient Without a Mandible; Dr. H. E. Jenkins, Gold Crowns; Dr. P. M. Crume, Hydrogen Flame; Dr. Henry Barnes, Method of Cleaning the

Teeth; Dr. M. G. Phillips, a Detachable Cusp for Steel Facing.

DAYTON'S HOSPITALITY. It was the universally expressed sentiment that Dayton's dentists had covered themselves with glory. The weather was fine until the last day. The hotels were ample and accommodating. The meeting hall was ideal, there was room for everything and the acoustics of the meeting hall were excellent, and there was no noise to disturb the proceedings. The clinicians had ample space and good facilities and every one had a chance to view the clinics. The exhibits were in a spacious room by themselves and were beautifully arranged. There were over fifty exhibits, and we have rarely seen them so well cared for. Both exhibitors and visitors were much pleased with this feature of the meeting.

The National Cash Register Co. invited the convention to inspect its plant, and also to a luncheon and moving picture show, all of which was most cordially enjoyed by a large number of the visiting dentists.

Dr. Custer promised a baloon trip on the afternoon of the last day, but unfortunately it rained and the event could not be witnessed by the dentists who were anxious to see Custer "blow up." If he didn't pull off this famous stunt for us he was one of the busiest men in Dayton, as he seemed determined to give everybody a good time. In fact all the Dayton dentists worked like "Trojans" to make the event successful and they certainly did an extraordinary bit of entertaining. In truth we never heard a complaint and we were on the look out for one all the time. If Cleveland entertains as well next year, it will tax their ingenuity as well as their energies to equal Dayton's example. While it seems like paying a big price for the privilege of entertaining, it will undoubtedly do much to make attending the state meeting more popular, and add much to the enthusiasm and esprit de corps of the profession in the state. This gain will justify the idea of passing around the meeting place rather than to confine it to Columbus.

## Figures and Tables

**Figure f1:**